# Physicians’ Relief Committee Report

**Published:** 1872-01-15

**Authors:** Chas. G. Smith

**Affiliations:** Sec’y.


### PHYSICIAN'S RELIEF COMMITTEE REPORT.

At a meeting of the physicians of Chicago, held on Dec. 25, at 797 Wabash avenue, to hear the report of the Medical Relief Committee, Dr. A. Fisher was chosen Chairman, and Dr. Chas. G. Smith, Secretary.

The Secretary of the Relief Committee, Dr. W. Hay, then read a report giving a detailed statement of the doings of the committee, the amount of money and books received and distributed, to whom the same had been given, and the correspondence with the profession in other parts of the country.

The treasurer of the committee, Dr. N. S. Davis, made the following report:

Whole amount of money received by the committee to date is $8,284.08. Of which $5,500 was received from the physicians of the city of New York; $1,238 from those of Brooklyn, N. Y.; $457 from those of Cincinnati; $100 from those of St. Louis; $253.08 from those of San Francisco; $200 from those of Washington, D. C.; $105 from those of Lowell, Mass.; $75 from the Rensselaer County Medical Society, New York, and the balance in small sums from individual contributions in different parts of the country.

It is proper to state that while the treasurer of the committee has received only $100 from St. Louis, the profession in that city contributed $900 more and sent it early after the fire in the hands of a committee from their own number, who distributed it to those among us most immediately suffering.

The whole amount actually distributed to physicians suffering from the effects of the fire, by the committee, to date, $6,520.

This has been given to 105 physicians and students, in sums varying from $30 to $180. There has also been paid to the society, for expense of printing circulars, express charges and postage, $17.50—leaving now in the hands of the treasurer, $1,745.58. There has also been received from two prominent members of the profession in Pennsylvania, twenty-two valuable medical works, which have also been distributed by the committee.

At the call of the meeting a full list of names of applicants for relief were given by the committee, including those assisted, and those whose claims had been rejected.

After a full discussion of the subject, the following resolutions were offered, the first by Dr. S. C. Blake, and the second by Dr. G. C. Paoli, and were unanimously adopted:

*Resolved*, That the further distribution of the funds still in the hands of the treasurer be left to the discretion of the committee.

*Resolved*, That the thanks of the physicians be given to the committee for the faithful and judicious discharge of the duties assigned to it.

Dr. N. S. Davis then offered the following resolutions, which were also adopted unanimously:

*Resolved*, That the cordial thanks of the profession in this city are tendered to their professional brethren in other parts of the country, particularly in New York, Brooklyn, St. Louis and Cincinnati, for the prompt and liberal aid they have given to those among us who have suffered severely by the fire.

*Resolved*, That such aid has been sufficient to meet the emergency, and that from this time forward we feel confident that the sufferers will be able to sustain themselves without further contributions from their brethren in other parts of the country.

On motion the meeting adjourned.

CHAS. G. SMITH, M. D., Sec'y.

